# Induction of Survival of Motor Neuron (SMN) Protein Deficiency in Spinal Astrocytes by Small Interfering RNA as an In Vitro Model of Spinal Muscular Atrophy

**DOI:** 10.3390/cells11030558

**Published:** 2022-02-05

**Authors:** Markus Leo, Linda-Isabell Schmitt, Michael Fleischer, Rebecca Steffen, Cora Osswald, Christoph Kleinschnitz, Tim Hagenacker

**Affiliations:** Department of Neurology and Center for Translational Neuro- and Behavioral Sciences (C-TNBS), University Hospital Essen, 45147 Essen, Germany; linda-isabell.schmitt@uk-essen.de (L.-I.S.); michael.fleischer@uk-essen.de (M.F.); rebecca.steffen@stud.uni-due.de (R.S.); cora.osswald@stud.uni-due.de (C.O.); christop.kleinschnitz@uk-essen.de (C.K.); tim.hagenacker@uk-essen.de (T.H.)

**Keywords:** spinal muscular atrophy, SMA, survival of motor neuron, SMN, siRNA, astrocytes, spinal cord, glia–neuron interaction, neuromuscular disorders

## Abstract

Spinal muscular atrophy (SMA) is a motor neuron disorder leading to progressive loss of ventral horn neurons resulting in muscle wasting. Here we investigate the contribution of spinal astrocytes to the pathogenesis of late-onset SMA forms using a mouse model. Furthermore, we generated SMA-like astrocytes using survival of motor neuron (SMN) siRNA transfection techniques. In the SMA mouse model, the activation of spinal astrocytes and the reduction of the inward rectifier potassium channel K_ir4.1_ and excitatory amino acid transporter 1 (EAAT1) were observed at postnatal day (P) 28, preceding the loss of spinal motor neurons appearing earliest at P42. Using SMA-like astrocytes, we could mimic the modulation of spinal astrocytes of the mouse model in a dish and perform electrophysiological assessments and functional assays. In SMA-like astrocytes, glutamate uptake was diminished due to a reduction in EAAT1. Furthermore, patch-clamp measurements revealed reduced potassium uptake into astrocytes with membrane depolarization. Additionally, exposure of healthy spinal motor neurons to a conditioned medium of SMA-like astrocytes resulted in increased firing frequency. These data demonstrate spinal astrocytes’ crucial role in the late-onset SMA forms’ pathogenesis.

## 1. Introduction

Astrocytes are the most common glial cell type in the central nervous system (CNS). Here, they regulate many physiological functions with importance for the correct functionality of the surrounding neurons. One of the key functions of astrocytes focuses on maintaining the homeostasis of excitatory substances such as extracellular potassium or the uptake and metabolism of the neurotransmitter glutamate from the synaptic cleft [1,2,3,4].

Astrocytes play a critical role in health and disease. Therefore, it is not surprising that misfunction of astrocytes contributes to many neurological disorders such as epilepsy, brain ischemia, Alzheimer’s disease (AD), Huntington’s disease (HD), or amyotrophic lateral sclerosis (ALS) [5,6,7,8,9]. For those disorders, dysfunctions in both the inward rectifier potassium channel K_ir4.1_ responsible for the uptake of extracellular potassium or the excitatory amino acid transporter 1 or 2 (EAAT1 or 2) contributing to the uptake of synaptically released glutamate, directly result in neuron hyperexcitability and potential apoptotic processes [10,11,12,13,14,15].

Spinal muscular atrophy (SMA) is a hereditary motor neuron disorder leading to progressive muscle wasting and weakness caused by the degeneration of ventral horn neurons in the spinal cord [16]. SMA is classified into different types regarding its severity and onset, being caused by a homozygous deletion or mutation of the survival of motor neuron 1 (*SMN1*) gene leading to a non-functional truncated SMN protein. The *SMN2* gene differs from the *SMN1* in a cytosine to thymine transition in exon 7 leading to the exclusion of exon 7 in the mRNA postprocessing, subsequently resulting in an unstable SMN protein [17,18]. The number of *SMN2* gene copies is known as the strongest disease modifier correlating negatively with the disease severity in SMA patients [19,20]. The SMN protein forms a complex with other proteins such as Gemin 2 to Gemin 7, which play an elementary role in assembling spliceosomal small nuclear ribonucleoproteins (snRNP), therefore being relevant for the pre-messenger ribonucleic acid (mRNA) [21].

Additional mechanisms underlying the pathology of SMA are yet not fully understood but described frequently. The differences between the early- and later-onset phenotypes could play a crucial role in understanding molecular mechanisms to identify new potential targets for SMN-independent therapeutic strategies.

In this study, we investigated the potential contribution of spinal astrocytes to the pathogenesis of a late-onset form of SMA. In studies of severe mouse models of SMA, the involvement of astrocytes in the pathogenesis of SMA has become evident. The restoration of SMN in motor neurons was less effective in the long-term rescue of an SMA mouse model than the restoration of SMN in spinal astrocytes [22,23]. Astrocytes, which lack SMN protein, showed increased activation markers such as glial fibrillary acidic protein (GFAP), differences in process length, abnormal calcium homeostasis, and abandoned support of motor neuron synapses in vitro [24,25,26]. Additionally, an increase in pro-inflammatory cytokines such as interleukin 6 (IL-6) and IL-1 beta, that astrocytes can express, was observed in post-mortem spinal cords of SMA-1 patients [27]. This data suggests a critical involvement of spinal astrocytes in SMA pathology.

Here, we focused on the expression of two proteins essential for spinal motor neurons physiology, the potassium channel K_ir4.1_ and the glutamate uptake protein EAAT1. Both astrocytic proteins and their contribution to SMA pathology have not been described in SMA mouse models or SMN-deficient spinal astrocytes so far. In addition, there is only one study based on human thalamus tissue of SMA type 1 patients showing a total loss of EAAT1 expression in astrocytes [28]. Therefore, we used two different types of research models. On the one hand, we used a mouse model, reflecting late-onset SMA, at two different time points during the pathogenesis and performed immunostaining. On the other hand, we generated SMA-like spinal astrocytes in a dish by siRNA transfection as an in vitro model to support the mouse model’s findings with further functional studies as patch-clamp measurements or functional assays. Afterward, we investigated the influence of SMA-like astrocytes on healthy spinal motor neurons’ excitability by calcium imaging.

Combining these two models may serve as a tool to identify the direct influence of SMN on a specific cell type such as spinal astrocytes. Thus, this could result in a simple model for functional studies to thoroughly examine the role of these cells in SMA pathogenesis.

## 2. Materials and Methods

### 2.1. Animals

SMN-deficient mouse model FVB.Cg-*Smn1^tm1Hung^* Tg(SMN2)2Hung/J (Jackson #005058), reflecting later-onset SMA, homozygote for the murine *SMN1* knockout, and the insert of human *SMN2* (4 copies) were purchased from Jackson Laboratory (Bar Habor, ME, USA) and bred in the Animal Research Lab of the University Hospital Essen. Male mice were used for spinal cord tissue harvesting at postnatal days (P) 28 and P42. Age-matched male FVB/N mice served as control.

For cell culture experiments, FVB/N mice were used. For isolating and culturing of spinal astrocytes, FVB/N mice at P25 were sacrificed. For the isolation and culturing of spinal motor neurons, FVB/N mice were timely paired, and spinal cord tissue of embryonic day (E) 13.5 embryos were harvested.

All animals were kept on a 14/10 h light/dark cycle with water and standard food pellets available ad libitum.

All experiments were conducted under the animal welfare guidelines of the University Duisburg Essen. Furthermore, the use of the SMA mouse model was approved by the State Agency for Nature, Environment and Consumer Protection (LANUV) in North Rhine-Westphalia (reference number 81-02.04.2020.A335).

### 2.2. Preparation of Spinal Cord Sections

Lumbar spinal cord tissue of FVB.Cg-*Smn1^tm1Hung^* Tg(SMN2)2Hung/J and control FVB/N mice were snap-frozen in liquid nitrogen and stored at −80 °C until usage. Twenty-micrometer Cryo-sections of spinal cords were prepared. Every fifth section of each spinal cord was assessed on one independent microscopy slide.

### 2.3. Isolation and Culture of Spinal Astrocytes from Wild-Type Mice

To isolate astrocytes from mouse spinal cords, animals were deeply anesthetized by isoflurane, and the spinal column was dissected. The whole spinal cord was removed by hydraulic extrusion and meninges were removed. Only the lumbar part of the spinal cord was used for astrocytes isolation. Spinal cord tissue was then chopped into a slurry using a razor blade and transferred to a 0.25% trypsin/EDTA solution (#25200056, Thermo Fisher Scientific, Dreieich, Germany) for 30 min at 37 °C. Enzymatic digestion was stopped by adding DMEM/F12 (#210410202, Thermo Fisher Scientific, Dreieich, Germany) containing 10% fetal bovine serum (FBS, #16140071, Thermo Fisher Scientific, Dreieich, Germany) to the solution. Afterward, tissue was mechanically triturated until a cell suspension was formed.

The cell suspension was brought to 10 mL using DMEM/F12 containing 10% FBS and 1% penicillin/streptomycin (P/S, #15140122, Thermo Fisher Scientific, Dreieich, Germany), placed into a 75 cm^2^ cell culture flask (T75), and incubated at 37 °C and 5% CO_2_. The next day, the medium was removed and replaced with a fresh culture medium. After that, the medium was removed and replaced every two days with fresh medium without AraC. After the cells reached approximately 65% confluency (10–14 days) and to ensure a single-cell layer, flasks were shaken on an orbital shaker (250 rpm at 37 °C, 5% CO_2_) overnight to remove microglia. Afterward, the culture medium was replaced, cells were scraped from the cell culture flask, counted, and placed on poly-d-lysine (PDL, Sigma-Aldrich, Taufkirchen, Germany)-treated glass coverslips in a 24-well plate (3500 cells per coverslip). After seven days in vitro (DIV) post replating, siRNA experiments were started.

### 2.4. Isolation and Culture of Spinal Motor Neurons from Embryonic Wild-Type Mice

For isolation of spinal motor neurons, lumbar parts of the spinal cords of E13.5 wild-type mouse embryos were harvested and meninges carefully removed. Afterward, the spinal cord tissue was enzymatically digested in 0.25% trypsin/EDTA solution for 30 min at 37 °C. After gentle mechanical disruption of the tissue, spinal cord cells were pre-plated on an uncoated plastic culture dish (10 cm) for 1 h at 37 °C to remove most of the non-neuronal cells as glial cells or fibroblasts. After this time, cell solution was removed from the dish and centrifuged at 500× *g* for 5 min. Next, the pellet was resuspended in DMEM:F12 1:1 containing 10% FBS, 1% P/S, and Glutamax (#35050061, Thermo Fisher Scientific, Dreieich, Germany). Next, cells were plated on PDL-coated (#P6407, Sigma Aldrich, Taufkirchen, Germany) glass coverslips (5000 cells per coverslip) and cultured at 37 °C and 5% CO_2_. At 1 DIV, the medium was changed to Neurobasal A medium (#10888022, Thermo Fisher Scientific, Dreieich, Germany) containing B27 (#17504044, Thermo Fisher Scientific, Dreieich, Germany), Glutamax, P/S, GDNF (#G-240, Alomone Labs, Jerusalem, Israel), BDNF (#B-250, Alomone Labs, Jerusalem, Israel), CNTF (#C-240, Alomone Labs, Jerusalem, Israel), and 1 µM AraC (#C6645, Sigma Aldrich, Taufkirchen, Germany). Afterward, the medium was then changed once a week. Cells were kept in culture until used in experiments.

### 2.5. siRNA Transfection of Cultured Spinal Astrocytes

The efficiency of this transfection method was evaluated using scrambled siRNA-FITC (#sc-36869, Santa Cruz, Dallas, TX, USA) and immunostaining for signal-positive cells.

In contrast to humans, mice and other rodents only have one *SMN* gene, called *SMN1*. Therefore, we used mouse-specific SMN1 siRNA (#SR408281, OriGene, Rockville, MD, USA) to induce SMN-deficiency in cultured spinal astrocytes. Cultured wild-type spinal astrocytes were transfected with SMN siRNA or scrambled siRNA (control) after 7 DIV.

Two hours before applying siRNA to the spinal astrocytes, the medium was removed and replaced by FBS-free medium (DMEM:F12 containing 1% P/S). Next, 10 nM of the SMN1 siRNA was mixed with 200 µM of Silence Mag (#SM11000, OZ Biosciences, Marseille, France) and incubated for 15 min at RT. After 15 min, the cells were incubated with the complex for 2 h on a magnetic plate at 37 °C and 5% CO_2_. Finally, the magnetic plate was removed, and cells were incubated until the next day. Then, the siRNA medium was replaced with fresh medium (DMEM/F12, 10% FBS, 1% P/S), and cells were kept in culture until use in experiments at DIV 10.

### 2.6. Immunostaining

Lumbar spinal cord sections, astrocyte or spinal motor neuron cultures were fixed in 4% Paraformaldehyde, washed, permeabilized (PBS, 0.1 *v/w* Triton X-100), and blocked (PBS, 5% bovine albumin serum). Primary antibodies for SMN (anti-SMN, rabbit, 1:200, #NBP2-76839, Novus Biologicals, Wiesbaden-Nordenstadt, Germany), motor neurons (anti-SMI-32, mouse, 1:400, #801701, BioLegend, San Diego, CA, USA or anti-ChAT, rabbit, 1:500, #AB143, Millipore, Darmstadt, Germany), GFAP (anti-GFAP, mouse, 1:500, #63893, Sigma Aldrich, Taufkirchen, Germany), K_ir4.1_ (anti-K_ir4.1_, rabbit, 1:500, #APC-035, Alomone Labs, Jerusalem, Israel), or EAAT1 (anti-EAAT1, rabbit, 1:500, #250113, Synaptic Systems, Göttingen, Germany) were diluted in blocking solution and incubated at 4 °C overnight. Sections were washed, and secondary antibodies (goat anti-rabbit, goat anti-mouse, 1:300, Dianova, Hamburg, Germany) and Dapi (1:1000, Sigma Aldrich, Taufkirchen, Germany) were diluted in blocking solution. Sections were incubated for 1.5 h at room temperature.

Images were obtained using a Zeiss Axio Observer.Z1 Apotome (Zeiss, Jena, Germany) fluorescence microscope, and Zeiss Zen software to determine the relative protein levels. All microscope settings such as laser intensity, exposure time, or contrast were kept the same for each protein to analyze.

Immunoreactivity was measured using Image J software (NIH, Bethesda, MD, USA) and protein fluorescence positive cells were selected using the freehand tool. The fluorescence intensity of each protein was measured and normalized against the background area in each image. In addition, the fluorescence intensity of SMA mouse tissue or SMN-deficient cultured astrocytes was normalized to control tissue or cells.

### 2.7. Electrophysiology

To isolate I_Kir4.1_ from cultured spinal astrocytes, whole-cell patch-clamp measurements were performed as described elsewhere [29]. Briefly, micropipettes were pulled from filament-containing borosilicate glass (1.5 mm OD × 0.86 mm ID × 100mm L, Bio-Medical Instruments, Zöllnitz, Germany) with a pipette puller (Sutter Instruments, Novato, CA, USA) to have a resistance of 3–7 MΩ. The internal solution (pipette solution) consists of 125 mM K-Gluconate, 2 mM CaCl_2_, 2 mM MgCl_2_, 10 mM EGTA, 10 HEPES, 0.5 mM Na-GTP, and 2 mM Na_2_ATP, pH with KOH (pH 7.2). While, the external solution (bath solution) consists of 14.4 mM NaHCO_3_, 5.9 mM KCl, 2. 5 mM MgSO_4_, 120.9 mM NaCl, 1.2 mM NaH_2_PO_4_, 2.5 mM CaCl_2_, 11.5 mM glucose (pH 7.4). The patch-clamp measurement was performed at a defined voltage, and spinal astrocytes were clamped at −70 mV with a depolarization range from -180 to 40 mV (in increments of 20 mV) to monitor the current–voltage relation curve (IV) of I_Kir4.1_. The membrane potential, current density, and cell capacitance were additionally recorded.

To evaluate the direct influence of K_ir4.1_ dysfunction on K_ir4.1_ current density and resting membrane potential of spinal astrocytes, cells were exposed to scrambled siRNA as described above. Afterward, astrocytes were exposed to 2 µM of specific K_ir4.1_ inhibitor VU0134992 (#6877/10, Tocris, Minneapolis, MN, USA) 24 h before starting patch-clamp experiments.

Patch-clamp protocols were evaluated using EPC10 amplifier, Patchmaster software (HEKA, Lambrecht, Germany), and Microsoft Excel. The Patchmaster software corrected “leakage currents” using an integrated P/4 protocol.

### 2.8. Measurement of ROS Production

CellRox assay (#C10444, Thermo Fisher Scientific, Dreieich, Germany) was performed following the manufacturer’s protocol to measure ROS production in cultured spinal astrocytes.

Images were obtained using a Zeiss Axio Examiner fluorescence microscope. The fluorescence intensity of SMN-deficient astrocytes was analyzed using Image J software (NIH, Bethesda, MD, USA) and normalized to control astrocytes intensity.

### 2.9. Glutamate Uptake Assay

For measuring glutamate uptake into SMN-deficient cultured spinal astrocytes, 200 µM glutamate was applied to the cells at 10 DIV. After 4 h of exposure to glutamate, the medium (astrocyte-conditioned medium) was collected and stored at −80 °C until use.

A lumbar spinal cord tissue lysate was prepared to measure glutamate level in spinal cord tissue of wild-type and SMA mice at P28.

According to the manufacturer’s protocol, the glutamate level of the astrocyte-conditioned medium and mice spinal cord tissue lysate was assessed by using a glutamate assay kit (#MAK004, SigmaAldrich, St. Louis, MO, USA).

### 2.10. Calcium Imaging

Calcium imaging measurements were performed to determine the impact of SMN-deficient spinal astrocytes on spinal motor neurons. Therefore, spinal motor neurons were isolated and cultured as described above. After 14 DIV, motor neurons were exposed to 10 µL of conditioned medium from scrambled or SMN siRNA transfected spinal astrocytes for 24 h. Afterward, spinal motor neuron cultures were incubated for 15 min with 1 µM calcium fluorescence dye Fluo-4 AM (#F14201, Thermo Fisher Scientific, Waltham, MA, USA) in FBS-free medium. Afterward, cells were washed with aCSF for 15 min. Spontaneous calcium spiking events were measured over a time period of 6 min. After 5 min, motor neurons were exposed to a pulse of potassium chloride (60 mM KCl).

Images were recorded at a frame rate of 0.5/s with a total exposure time of 100 ms over 6 min using a Zeiss Axio Examiner fluorescent microscope. In addition, active cells were detected and calcium-signals recorded with the open-source software suite2p [30].

Spontaneous spiking events per second (spiking frequency) were analyzed using MATLAB (The Math Works, Inc., Natick, MA, USA, MATLAB. Version 9.10.0.1710957 (R2021a), 2021). For analysis, only cells responding to the KCl pulse were considered. The spiking frequencies of motor neurons exposed to the conditioned medium of SMN-deficient astrocytes (cond.) were compared to those exposed to the conditioned medium of scrambled siRNA transfected astrocytes (control).

### 2.11. Statistics

Mann–Whitney-U or one-way ANOVA was used for the statistical analysis of data from mouse spinal cord tissue. In addition, statistical analysis of cell culture experiments was performed by using Student’s *t*-test.

Significances were defined at a value of *p* < 0.05. All values are given as means ± “Standard Error of Mean” (SEM).

## 3. Results

### 3.1. Mouse Model of Late-Onset SMA Shows SMN Deficiency and Loss of Spinal Motor Neurons

The hallmark characteristics of SMA are the reduction of SMN protein with subsequent loss of spinal motor neurons. Therefore, we performed immunostaining of spinal cord slices against SMN protein and SMI-32 as a marker for spinal motor neurons to investigate these characteristics.

**Figure 1:**
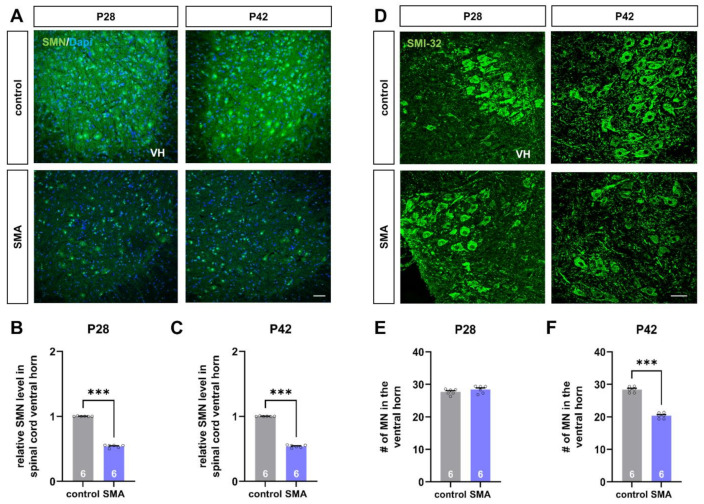
Deficiency of SMN protein and loss of spinal motor neurons in a mouse model of late-onset SMA. (**A**) Immunostaining of SMN protein (green) in spinal cord slices of control and SMA mice at P28 and P42. Nuclear DNA was stained with Dapi (blue). (**B**,**C**) SMN protein level was reduced in the spinal cord ventral horn of SMA mice at P28 and P42, compared to control mice at the same age (***, *p* < 0.001). (**D**) Immunostaining of spinal motor neurons (SMI-32, green) in spinal cord slices of control and SMA mice at P28 and P42. (**E**,**F**) The number of spinal motor neurons was reduced in SMA mice at P42, compared to control animals (***, *p* < 0.001). No loss of spinal motor neurons was observed at P28 (*p* > 0.05). VH = ventral horn. *n* = 6 animals (see bars). Three slices per spinal cord were investigated. Each data point reflects the mean of those three spinal cord slices. Scale bar: A = 20 µm; B = 50 µm.

The late-onset SMA mouse model used here showed a reduced SMN protein level in the spinal cord ventral horn at P28 and P42, compared to control mice (*p* < 0.001) (Figure 1A–C). In addition, loss of motor neurons in the SMA mouse model was observed at P42 (*p* < 0.001) but not at P28 (*p* > 0.05) (Figure 1D–F).

### 3.2. Mouse Model of Late-Onset SMA Shows Alteration in Astrocyte-Specific Proteins before the Loss of Motor Neurons

To investigate the potential contribution of spinal astrocytes to SMA pathogenesis, we examined the protein level of astrocyte-specific proteins with importance to motor neuron function in SMA and control mice before the first loss of spinal motor neurons at the age of P28.

**Figure 2:**
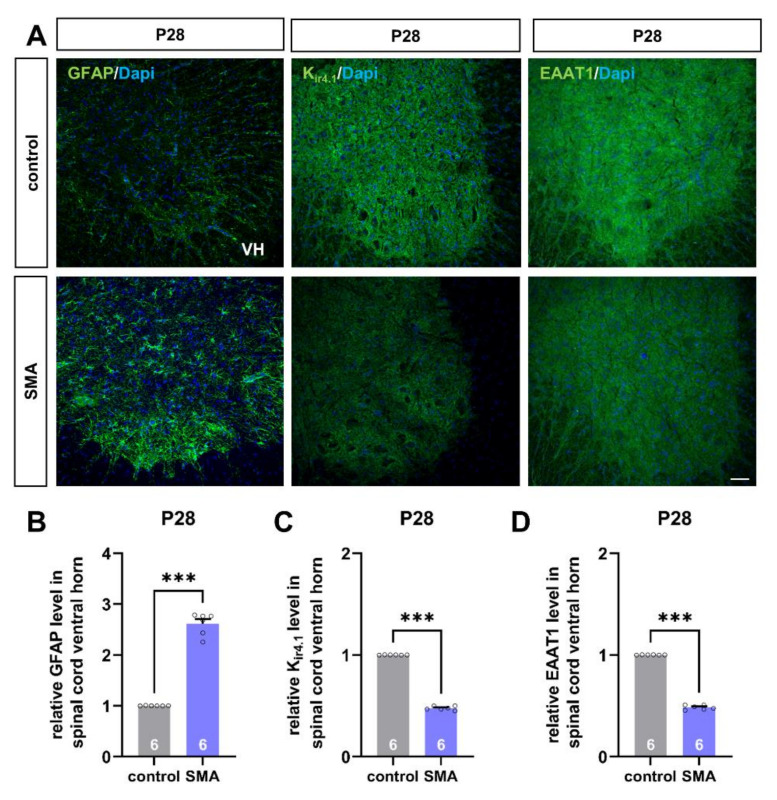
Spinal astrocytes proteins are altered in a mouse model of late-onset SMA before the first loss of motor neurons. (**A**) Immunostaining of astrocyte-specific proteins such as GFAP (green), K_ir4.1_ (green), and EAAT1 (green) in spinal cord slices of SMA and control mice at P28. Nucleic DNA was stained with Dapi (blue). (**B**) The relative GFAP level in SMA mice spinal cord ventral horns was increased compared to control mice (***, *p* < 0.001). (**C**,**D**) The relative level of K_ir4.1_ and EAAT1 was reduced in SMA mice, compared to control animals (***, *p* < 0.001). VH = ventral horn. *n* = 6 animals (see bars). Three slices per spinal cord were investigated. Each data point reflects the mean of those three spinal cord slices. Scale bar: 20 µm.

Immunostaining of spinal cord slices showed modulation of protein levels of GFAP, K_ir4.1_, and EAAT1 in SMA mice (Figure 2A). We observed an increased protein level of GFAP as a sign of increased astrocyte reactivity in SMA mice, compared to control mice of the same age (*p* < 0.001) (Figure 2B).

In contrast to GFAP expression, the protein level of K_ir4.1_ was reduced in mice suffering from SMA (*p* < 0.001) (Figure 2C).

Similar to this finding, the level of glutamate uptake protein EAAT1 was reduced under SMA conditions as well (*p* < 0.001) (Figure 2D).

### 3.3. SMA-like Spinal Astrocytes Can Be Generated In Vitro by SMN siRNA Transfection

Data gained from the late-onset SMA mouse model suggest a critical role for spinal astrocytes in the pathogenesis of SMA.

**Figure 3:**
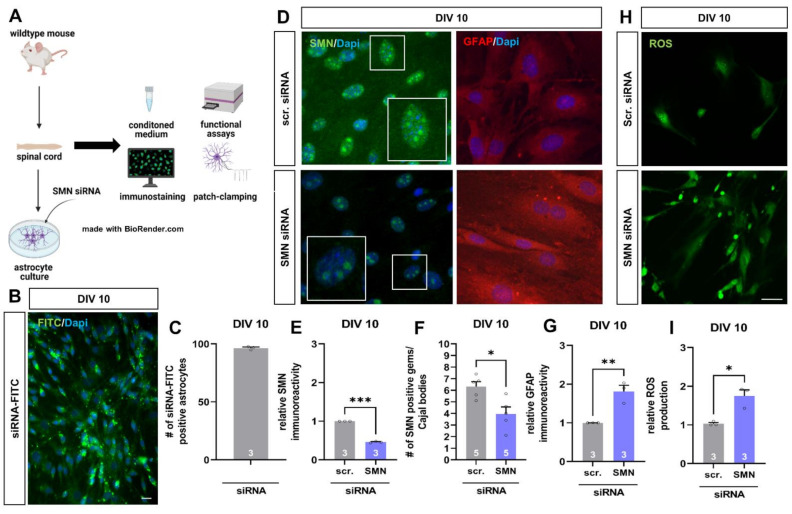
SMN-deficient spinal astrocytes can be generated by SMN siRNA in vitro, showing similar protein alteration as spinal astrocytes in the late-onset SMA mouse model. (**A**) Schematic drawing of the generation of SMA-deficient spinal astrocyte cultures by SMN siRNA and performed experiments. (**B**) Transfection of wild-type astrocyte cultures with scrambled siRNA-FITC (green) by magnetic transfection method. Nuclear DNA was stained with Dapi (blue). (**C**) Cultured wild-type astrocytes were successfully transfected with siRNA-FITC using the magnetic transfection method. (**D**) Immunostaining of wild-type astrocyte cultures transfected with scrambled (scr.) or SMN siRNA against SMN protein (green) and GFAP (red) at DIV 10. In addition, nuclear DNA was stained with Dapi (blue). (**E**) Transfection of wild-type spinal astrocytes with SMN siRNA resulted in a reduction of SMN protein similar to the level observed in the juvenile SMA type III mouse model (***, *p* < 0.001). (**F**) SMN positive gems/Cajal bodies in the nucleus of astrocytes were reduced by transfection with SMN siRNA (*, *p* < 0.05). Furthermore, an increased number of GFAP-positive aggregate-like structures were observed in SMN-deficient astrocytes (white arrows). (**G**) SMN-deficiency in spinal astrocytes induced by SMN siRNA resulted in increased GFAP level (**, *p* < 0.01). (**H**) Staining of ROS production (green) in wild-type astrocytes transfected with scrambled or SMN siRNA at DIV 10. (**I**) SMN-deficient astrocytes showed an increased production of ROS compared to astrocytes transfected with scrambled siRNA (*, *p* < 0.05). *n* = 3–5 independent experiments (see bars). For each experiment > 50 cells were analyzed. Scale bar: B = 50 µm; F = 20 µm.

To examine the direct influence of reduced SMN protein level on spinal astrocytes in detail, we generated SMN-deficient astrocytes in vitro to mimic SMA-like astrocytes by using cells of wild-type mice and magnetic transfection with SMN siRNA. Afterward, we investigated the reproducibility of SMN reduction on spinal astrocytes previously observed in the mouse model. Furthermore, we performed functional assays and patch-clamp electrophysiology (Figure 3A).

For evaluating the transfection efficiency of the magnetic transfection method, cultured wild-type spinal astrocytes were transfected with scrambled siRNA coupled to FITC, resulting in 96.48 ± 0.86% of FITC-signal positive cells (Figure 3B,C).

When cultured wild-type spinal astrocytes were transfected with scrambled or SMN siRNA at DIV 7, we performed immunostaining against SMN protein and GFAP at DIV 10 (Figure 3D). Spinal astrocytes transfected with SMN siRNA showed a reduced protein level of SMN compared to cells transfected with scrambled siRNA (*p* < 0.001) (Figure 3E).

Survival of motor neuron proteins is not only present in the cytoplasm of the cells but also in the nucleus within the so-called gems/Cajal bodies. The number of SMN positive gems/Cajal bodies in the nucleus of SMN siRNA transfected astrocytes was reduced compared to scrambled siRNA transfected cells (*p* < 0.05) (Figure 3F).

In SMN-deficient astrocytes, the protein level of GFAP at DIV 10 (*p* < 0.01) was increased, similar to the late-onset SMA mouse model. Moreover, GFAP-positive aggregate-like structures were noticeable in SMN-deficient astrocytes (Figure 3G).

Overexpression of GFAP is a sign of increased reactivity of astrocytes. To further examine the activity of SMN-deficient astrocytes, we measured the production of reactive oxygen species (ROS) at DIV 10 (Figure 3H). Here, ROS production increased when astrocytes were transfected with SMN siRNA compared to cells transfected with scrambled siRNA (Figure 3I).

### 3.4. SMN-Deficient Spinal Astrocytes Show Altered Expression and Function of K_ir4.1_ Channel Protein

One of the major functions of spinal astrocytes is to maintain the homeostasis of excitatory substances such as extracellular potassium by taking it up via channels such as the potassium channel K_ir4.1_.

**Figure 4:**
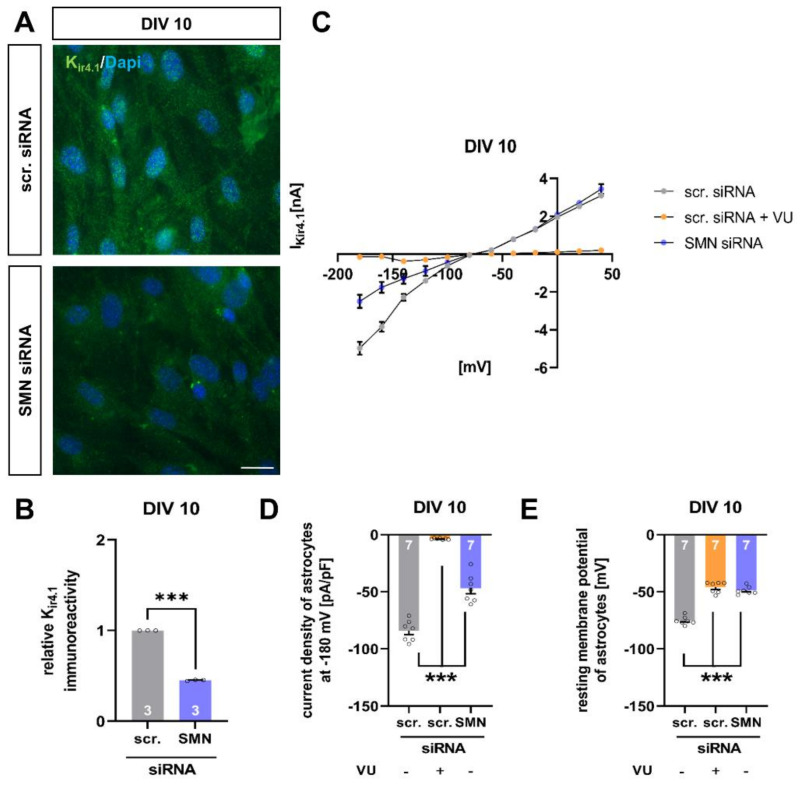
SMN-deficient spinal astrocytes show a reduction in K_ir4.1_ protein level and modulation in electrophysiological properties. (**A**) Immunostaining of wild-type astrocyte cultures transfected with scrambled (scr.) or SMN siRNA against K_ir4.1_ protein (green) at DIV 10. Nuclear DNA was stained with Dapi (blue). (**B**) SMN-deficient spinal astrocytes showed reduced protein levels of K_ir4.1_, compared to astrocytes transfected with scrambled siRNA (***, *p* < 0.001). (**C**) Raw trace of current–voltage (IV) curve of K_ir4.1_ channel protein in cultured spinal astrocytes transfected with scrambled or SMN siRNA or scrambled siRNA + K_ir4.1_ inhibitor VU. SMN-deficient astrocytes showed reduced current as a sign of reduced potassium uptake into spinal astrocytes. A similar effect was observed when VU inhibited the Kir4.1 function. (**D**) Current density of K_ir4.1_ was reduced in SMN-deficient spinal astrocytes, compared to astrocytes transfected with scrambled siRNA (***, *p* < 0.001). When K_ir4.1_ function was inhibited in wild-type astrocytes exposed to scrambled siRNA, the current density was reduced (***, *p* < 0.001). (**E**) Astrocytes transfected with SMN siRNA showed depolarized resting membrane potential (***, *p* < 0.001). A similar result was observed when K_ir4.1_ function was inhibited by VU (***, *p* < 0.001). *n* = 3 independent experiments for immunostaining (see bars). For each experiment > 50 cells were analyzed. *n* = 7 cells per electrophysiological measurement (see bars). Scale bar: 50 µm.

Immunostaining of spinal cord tissue from the late-onset SMA mouse model revealed a reduction of K_ir4.1_ protein level at P28. Furthermore, when cultured wild-type spinal astrocytes were transfected with SMN siRNA, the K_ir4.1_ protein level was reduced as well (*p* < 0.001), therefore mimicking the in vivo situation under SMA conditions (Figure 4A,B).

Additionally, we performed patch-clamp measurements of wild-type spinal astrocytes transfected with scrambled or SMN siRNA. Here, SMN deficient astrocytes showed reduced uptake of potassium ions resulting in a decreased current density of K_ir4.1_ (*p* < 0.001). A similar effect was observed when spinal astrocytes were exposed to scrambled siRNA, and K_ir4.1_ function was inhibited by specific blocker VU (*p* < 0.001) (Figure 4C,D).

Furthermore, the reduction of K_ir4.1_ protein level by SMN-deficiency resulted in a depolarization of the astrocytes’ resting membrane potentials (*p* < 0.001). Additionally, the inhibition of K_ir4.1_ function in spinal astrocytes exposed to scrambled siRNA by VU resulted in a depolarization of the resting membrane potential (*p* < 0.001) (Figure 4E).

### 3.5. SMN-Deficient Spinal Astrocytes Show Altered Expression of EAAT1 Protein and Reduced Uptake of Glutamate

Besides maintaining potassium homeostasis, another crucial function of spinal astrocytes is to keep the extracellular glutamate level low by taking it up via transport proteins such as EAAT1.

**Figure 5:**
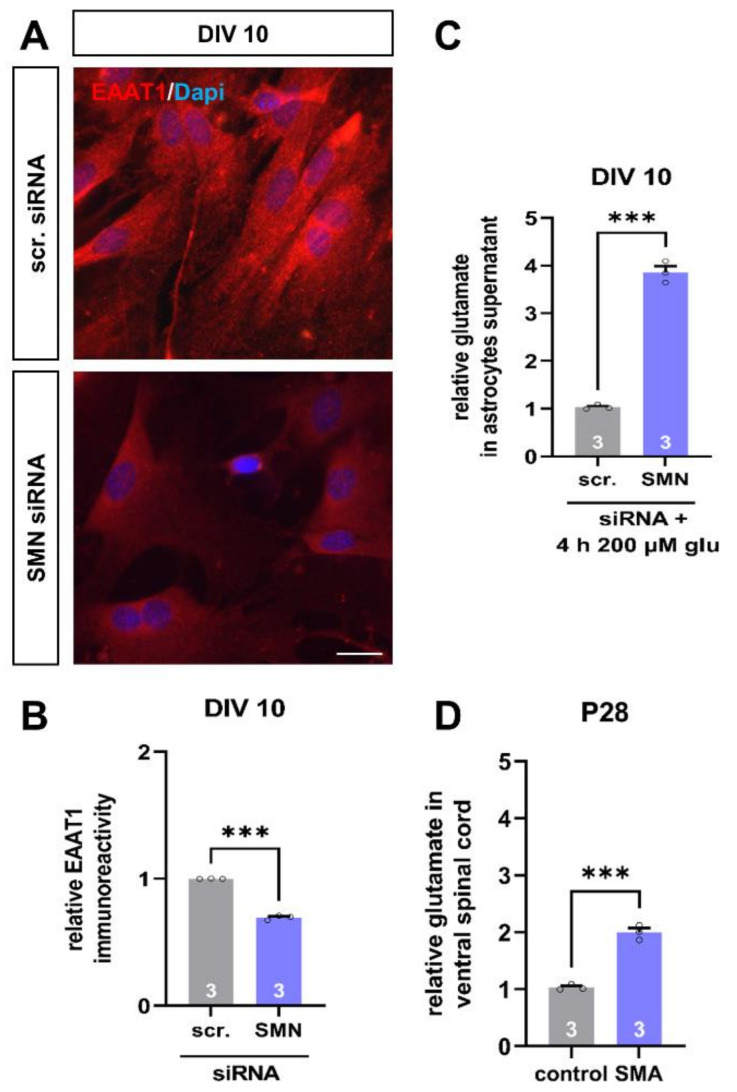
SMN-deficient spinal astrocytes show a reduction in EAAT1 protein level and reduced uptake of glutamate. (**A**) Immunostaining of wild-type astrocyte cultures transfected with scrambled (scr.) or SMN siRNA against EAAT1 protein (green) at DIV 10. In addition, nuclear DNA was stained with Dapi (blue). (**B**) SMN-deficient spinal astrocytes showed reduced protein levels of EAAT1, compared to astrocytes transfected with scrambled siRNA (***, *p* < 0.001). (**C**) When astrocytes were exposed to 200 µM of glutamate (glu) for 4 h, SMN-deficient astrocytes showed reduced uptake of the transmitter, compared to astrocytes transfected with scrambled siRNA (***, *p* < 0.001). (**D**) Ventral horn spinal cord tissue of juvenile SMA type III mouse model showed an increased glutamate level at P28, compared to control animals (***, *p* < 0.001). *n* = 3 independent experiments for immunostaining (see bars). For each experiment > 50 cells were analyzed. *n* = 3 independent experiments per functional measurement (see bars). Glutamate measurements of spinal cord tissue were from three individual animals. Scale bar: 50 µm.

When we transfected wild-type astrocytes with SMN siRNA, immunostaining revealed a reduction in EAAT1 protein level at DIV 10 (*p* < 0.001) (Figure 5A,B). Thus, this is in line with the results obtained from the spinal cord tissue of our SMA mouse model at P28.

In addition to immunostaining, we performed glutamate uptake experiments. Therefore, we transfected wild-type astrocytes with scrambled or SMN siRNA and exposed them to 200 µM of glutamate for 4 h at DIV 10. Afterward, we collected the medium and measured the remaining glutamate level. Here, we observed an increased glutamate level in the medium of SMN deficient astrocytes, compared to astrocytes transfected with scrambled siRNA, assuming a reduced uptake of glutamate into cells due to the lack of EAAT1 (*p* < 0.001) (Figure 5C).

We additionally measured the glutamate level in spinal cord tissue of control and SMA mice at P28. Here, we also observed an increase in late-onset SMA mice glutamate level compared to control mice (*p* < 0.001).

### 3.6. SMN-Deficient Spinal Astrocytes Show Altered Expression of EAAT1 Protein and Reduced Uptake of Glutamate

To examine a critical contribution of spinal astrocytes to the pathogenesis of SMA, we isolated and cultured spinal motor neurons from wild-type mouse embryos at E13.5.

**Figure 6:**
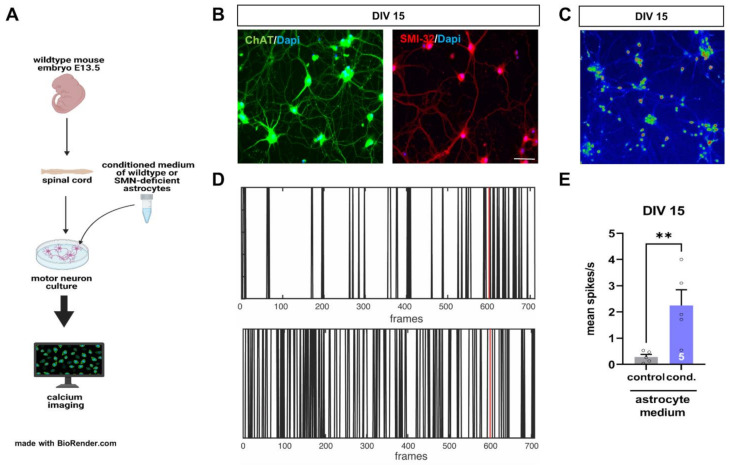
Conditioned medium of SMN-deficient astrocytes leads to hyperactivity of cultured spinal motor neurons of wild-type mice. (**A**) Schematic drawing of experimental design. Motor neurons were isolated from E13.5 embryos of wild-type mice. Cultured motor neurons were exposed to a conditioned medium of astrocytes transfected with scrambled or SMN siRNA for 24 h. Afterward, calcium imaging measurements were performed. (**B**) Immunostaining of cultured motor neurons at DIV 15. Neurons expressed typical markers of motor neurons such as ChAT (green) or SMN-32 (red). In addition, nuclear DNA was stained with Dapi (blue). (**C**) Image with Fluo-4 AM stained motoneurons at DIV 15 in LUT (16 colors). (**D**) Image of spiking events over the recording period. The red line indicates the application of potassium pulse (60 mM KCl). (**E**) Motor neurons exposed to a conditioned medium of SMN-deficient astrocytes showed an increased number in their spiking frequency (**, *p* < 0.01). *n* = 5 motor neurons measured per experiment (see bars). Scale bar: 20 µm.

After DIV 14, cultured motor neurons were exposed to a conditioned medium from astrocytes transfected with scrambled (control) or SMN siRNA (cond.) for 24 h. Afterward, calcium imaging measurements with Fluo-4 dye were performed, and spiking events per second were counted at DIV 15 (Figure 6A).

Immunostaining of cultured spinal cord neurons showed the expression of the motor neuron markers ChAT and SMI-32 in these cells at DIV 15, identifying them to be spinal motor neurons (Figure 6B).

Calcium imaging measurements showed increased spiking frequency (spikes/s) in motor neurons exposed to a conditioned medium from astrocytes transfected with SMN siRNA for 24 h, compared to neurons exposed to conditioned medium from scrambled siRNA transfected astrocytes (Figure 6C–E).

## 4. Discussion

Our findings suggest a critical role of spinal astrocytes in the pathogenesis of late-onset SMA forms by influencing the excitability of spinal motor neurons. Furthermore, we described a simple method to investigate the direct influence of SMN-deficiency on spinal astrocytes and their functionality using a cell culture-based in vitro approach and siRNA transfection technique. Here, we generated SMA-like astrocytes in a dish, showing similar protein alteration as spinal astrocytes of the SMA mouse model.

The mouse model reflects late-onset forms of SMA. However, in contrast to mouse models reflecting severe early-onset SMA, these mice suffer from milder motor deficits, are fertile, and present a normal life span [31,32].

We here present typical hallmark characteristics of SMA in a mouse model of the late-onset form. While the reduced SMA protein level remained constant in the SMA mice at both time points, P28 and P42, motor neuron loss was observed only at the later time point as a typical sign of slow progressive degeneration of spinal motor neurons in late-onset SMA compared to more severe types of SMA.

Interestingly, the potential modulation of astrocyte-specific proteins with importance for motor neuron function such as EAAT1 or K_ir4.1_ occurred before the first loss of spinal motor neurons appeared at P28. Thus, this is in line with the findings of McGivern et al. who showed the activation of spinal astrocytes before the first motor neurons got lost in a mouse model of a more severe SMA model [24]. In general, different models such as iPSC-derived astrocytes from SMA patients or astrocytes isolated from the SMN∆7 mouse model gave evidence for the activation of GFAP and changes in astrocytes morphology. In the human spinal cord from SMA type 1 patients, both astrogliosis and glial activation were observed, as shown by increased GFAP and pro-inflammatory cytokines expression, i.e., IL-6 and IL-1beta [27,33].

Immunostaining of spinal cord tissue from a late-onset SMA mouse model revealed the reduction of two astrocytic proteins with importance for motor neuron functionality. The glutamate uptake protein EAAT1 and potassium channel K_ir4.1_, responsible for the homeostasis of the extracellular potassium concentration, were reduced before the first loss of spinal motor neurons. The EAAT1 reduction and the resulting increased glutamate level in the spinal cord tissue of SMA mice demonstrate a potential role of glutamate-mediated excitotoxicity contributing to the loss of spinal motor neurons in SMA pathology. Glutamate excitotoxicity has been described in neurological disorders such as ischemia, epilepsy, or ALS [5,34]. Persistent increase in glutamate concentration within the spinal cord may occur through enhanced release from presynaptic neurons or surrounding astrocytes or be mediated by the reduced uptake into the astrocytes by dysfunction of glutamate-uptake proteins EAAT1 or EAAT2 [5,6,7,8,35,36]]. In ALS models and spinal cord tissue from ALS patients, EAAT2 protein was downregulated [5,7,35,37]].

Furthermore, the reduction of K_ir4.1_ channel protein in spinal cord tissue of our SMA mouse model occurred before the loss of spinal motor neurons appeared. The K_ir4.1_ protein plays a crucial role in the uptake of extracellular potassium from the synaptic cleft. Therefore, reduction or dysfunction of the K_ir4.1_ channel protein can lead to an excitatory increase in extracellular potassium resulting in depolarization of the close-by neurons [38,39,40,41,42].

Due to the challenges during the in vivo studies associated with investigating pathological mechanisms in neurodegenerative disorders, especially when evaluating a specific effect on a particular cell type, we decided to generate SMA-like astrocytes in vitro to mimic in vivo SMA conditions and investigate the direct impact of SMN reduction on spinal astrocytes in more detail. Therefore, we used spinal astrocytes of wild-type mice and transfected them with SMN siRNA to induce SMN-deficient astrocytes in a dish.

We were able to show that it is possible to generate SMA-like astrocytes using the magnetic transfection technique. Astrocytes transfected with SMN siRNA showed similar protein alterations as we observed for the mouse model. Furthermore, transfected astrocytes showed an almost identical reduction of SMN protein level as those cells within the spinal cord tissue of the late-onset SMA mouse model, proving the successful in vitro mimicking of the SMA mouse model on the astrocytic level. Furthermore, we observed a similar activation of spinal astrocytes transfected with SMN siRNA compared to the in vivo situation of the mouse model. Additionally, SMA-like astrocytes showed GFAP positive aggregates within their cell bodies, which were not apparent in scrambled siRNA transfected astrocytes. These aggregates could be some form of lysosomes containing GFAP that the astrocytes want to remove due to their overexpression. Further studies should address this by staining for lysosomal markers or alpha-B crystallin.

Our results regarding the activity of spinal astrocytes after siRNA transfection or in the mouse model were substantiated by measurements of ROS in SMN-like astrocytes in vitro, proving strong activation of these cells directly linked to the SMN protein reduction. A potential trigger of oxidative stress in astrocytes can be a modulation of intracellular calcium concentration [43]. In different studies, the effect of SMN on regulating calcium homeostasis in astrocytes was shown. In SMA iPSC-derived astrocytes, an increased basal calcium level could be observed compared to control iPSC-derived astrocytes [24]. On the other hand, a lower basal calcium level was measured in SMA astrocytes from SMN∆ 7 mice. However, stimulation with potassium chloride (KCl) increased the calcium peak in SMA astrocytes significantly compared to WT astrocytes [25]. This data suggests that SMN deficiency alters intracellular calcium signaling in astrocytes, leading to the activation of pathophysiological processes such as increased ROS production.

Our in vitro model of SMA-like astrocytes also showed the EAAT1 and K_ir4.1_ protein reduction observed in the late-onset SMA mouse model. In addition to the immunostaining, we performed patch-clamp measurements of the K_ir4.1_ channel protein and a glutamate uptake assay in SMA-like astrocytes after SMN siRNA transfections. Patch-clamp measurements of K_ir4.1_ in SMA-like astrocytes revealed a decreased current density due to protein level reduction leading to reduced potassium uptake as shown by the currently reduced peak in the IV curve, compared to scrambled siRNA treated cells. This data supports the findings of the protein level reduction observed in the mouse model and further substantiates them with functional measurements.

When SMA-like astrocytes were exposed to glutamate in vitro for 4 h, a higher glutamate level was found in the medium compared to astrocytes transfected with scrambled siRNA. Therefore, this supports the decreased uptake of glutamate due to the reduction of EAAT1 protein levels in these cells. These findings are also in line with the observed effects in the mouse model, proving one more time the usability of the in vitro cell model for investigating the role of astrocytes in late-onset SMA. Furthermore, it is broadly described that a critically increased level of the excitatory transmitter glutamate contributes to neuronal death by excessive calcium overload resulting in caspase activation and apoptosis [44,45,46,47,48], impacting motor neuron degeneration in SMA.

We also investigated the direct influence of SMA-like astrocytes on the excitability of healthy spinal motor neurons. When motor neurons were exposed to a conditioned medium of SMA-like astrocytes, we observed an increase in the spiking frequency by calcium imaging measurements suggesting the release of substances from SMA-like astrocytes as cytokines or glutamate potentially. Increased calcium influx into the spinal motor neurons could contribute to their degeneration during SMA pathogenesis by activating calcium-mediated death signaling pathways [49,50]. In the conditioned medium of SMN deficient astrocytes isolated from SMN∆7 mice, abnormal levels of glia-secreted factors were detected. Furthermore, when cultured motor neurons were exposed to this astrocyte-conditioned medium, a reduction in the length of their neurites was observed [51]. To our knowledge, this is the first time that K_ir4.1_ and EAAT1 modulation is described for late-onset SMA as a potential driving force of motor neuron degeneration. Furthermore, to our knowledge, this is the first study showing functional and electrophysiological measurements of spinal astrocytes in a model of SMA. The data gained here show a critical role for spinal astrocytes in the pathogenesis of SMA and make them an attractive target for further research.

## 5. Conclusions

Combining in vitro generated SMA-like cells such as astrocytes, and SMA mouse tissue could be a powerful tool for investigating the direct impact of lacking SMN protein on a specific cell type. Similarly, we here aimed to thoroughly contribute to the pathological progress of SMA that may potentially lead to functional assays or treatment validation experiments for further identifying novel therapeutic strategies.

Additionally, in the sense of 3Rs, reducing the number of experimental animals is critical. With the presented method, data can be easily and quickly generated from cell cultures of a single cell type reflecting SMA conditions, gained from fewer animals than needed for in vivo interventions. Hence, this should ultimately result in fewer animal numbers during in vivo experimentation.

## Data Availability

The data supporting the study findings are available on request from the corresponding author (M.L.).
